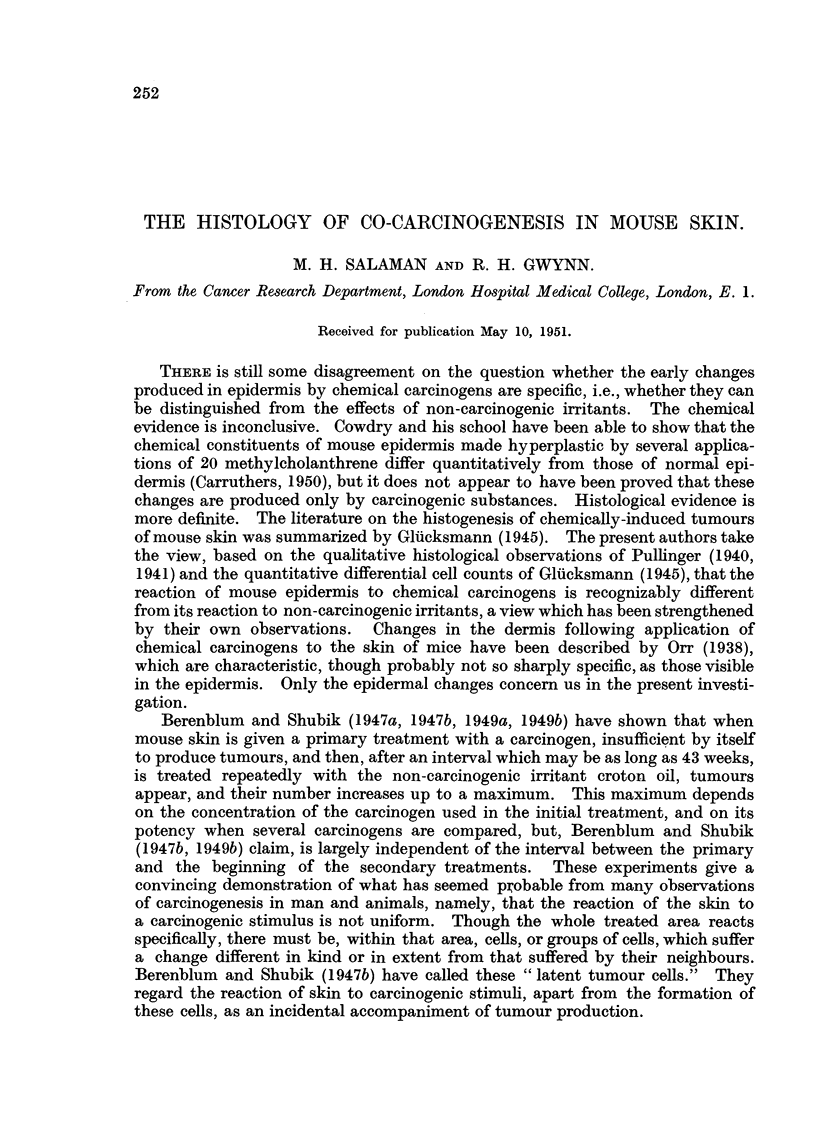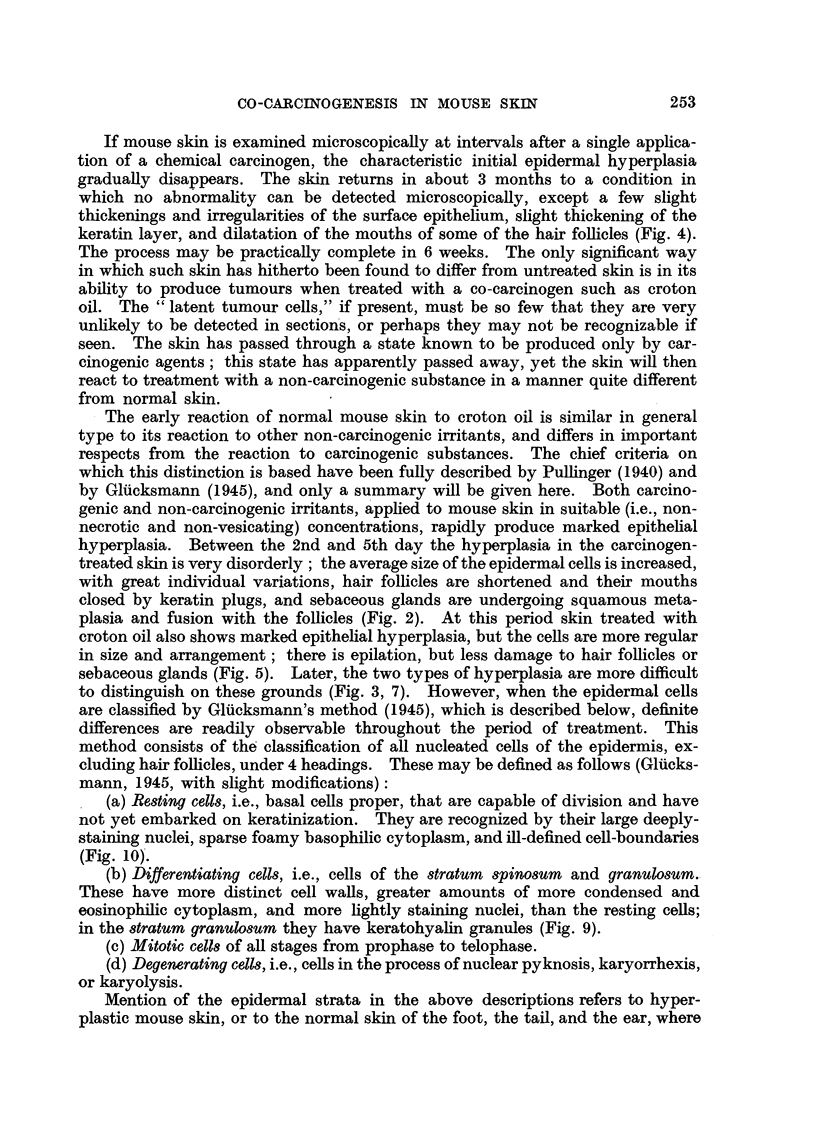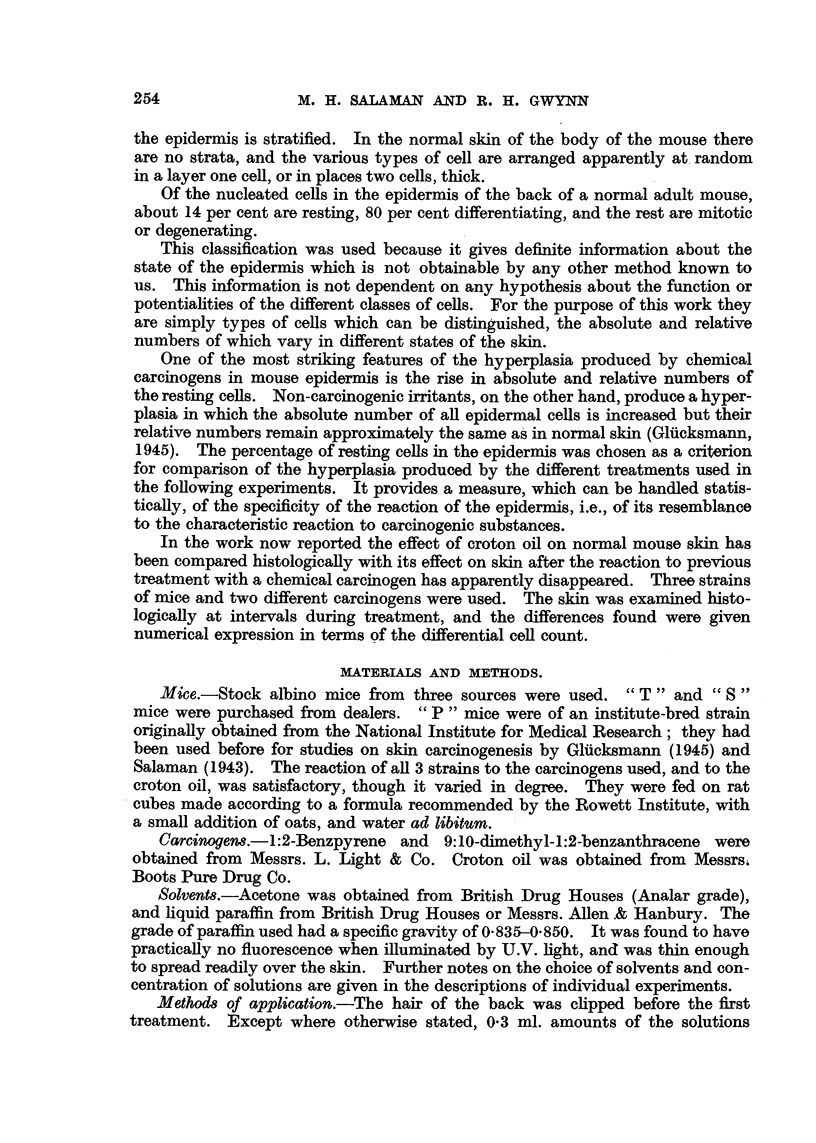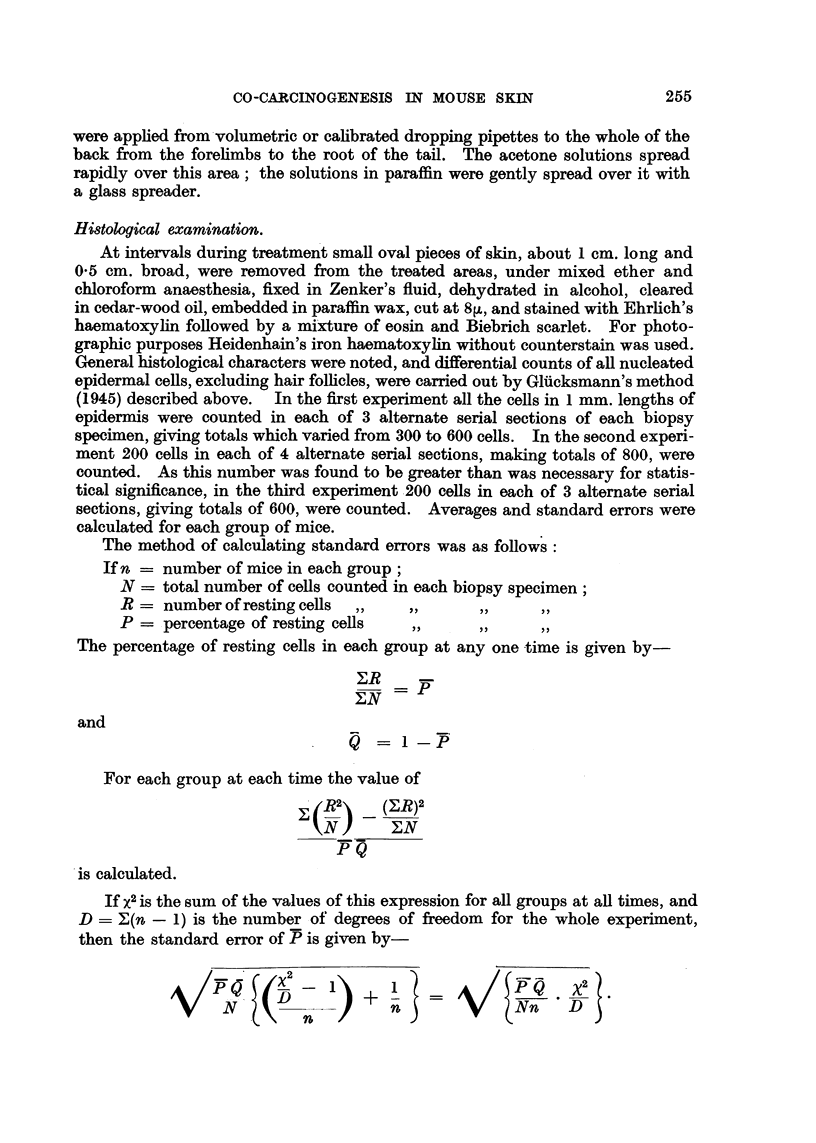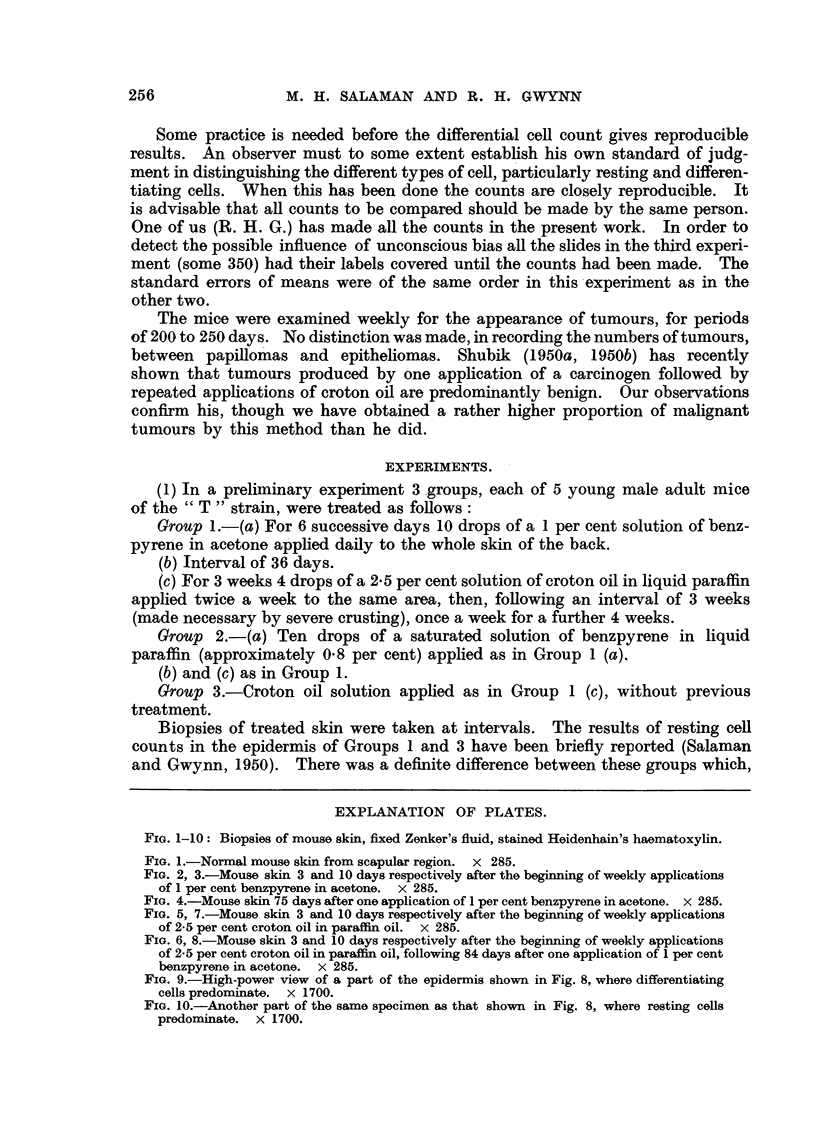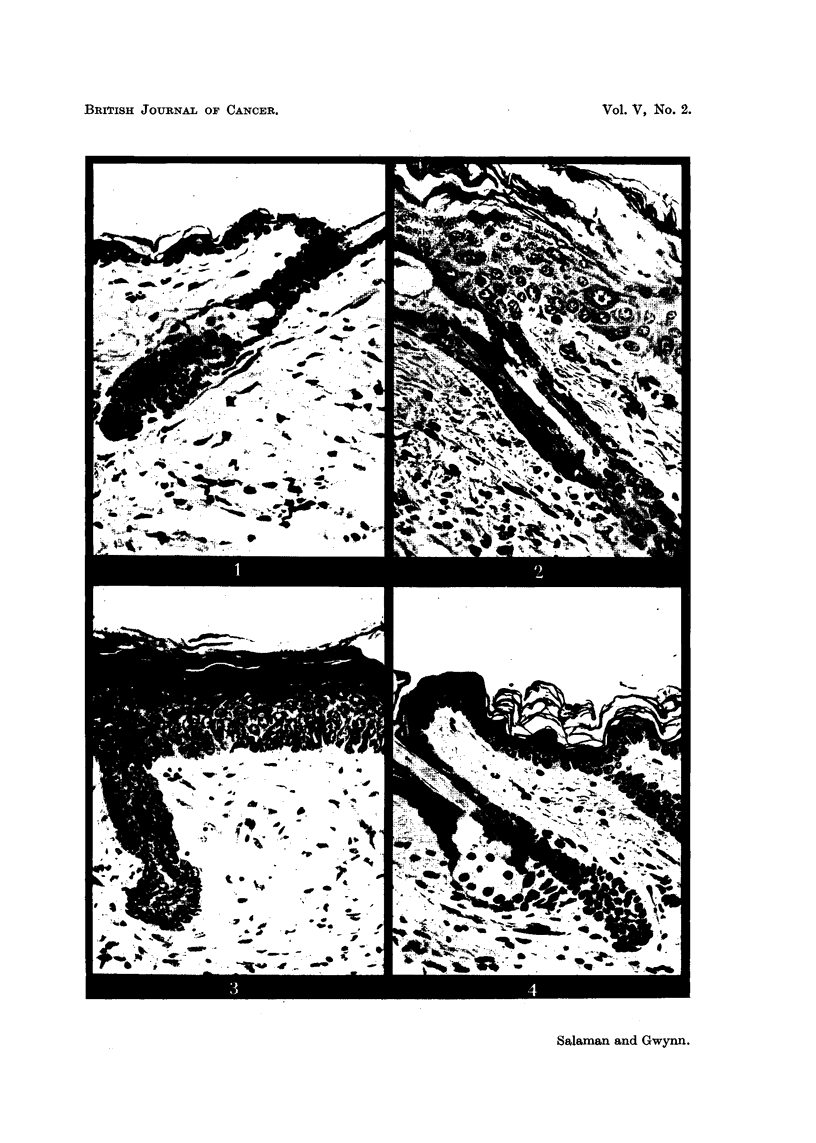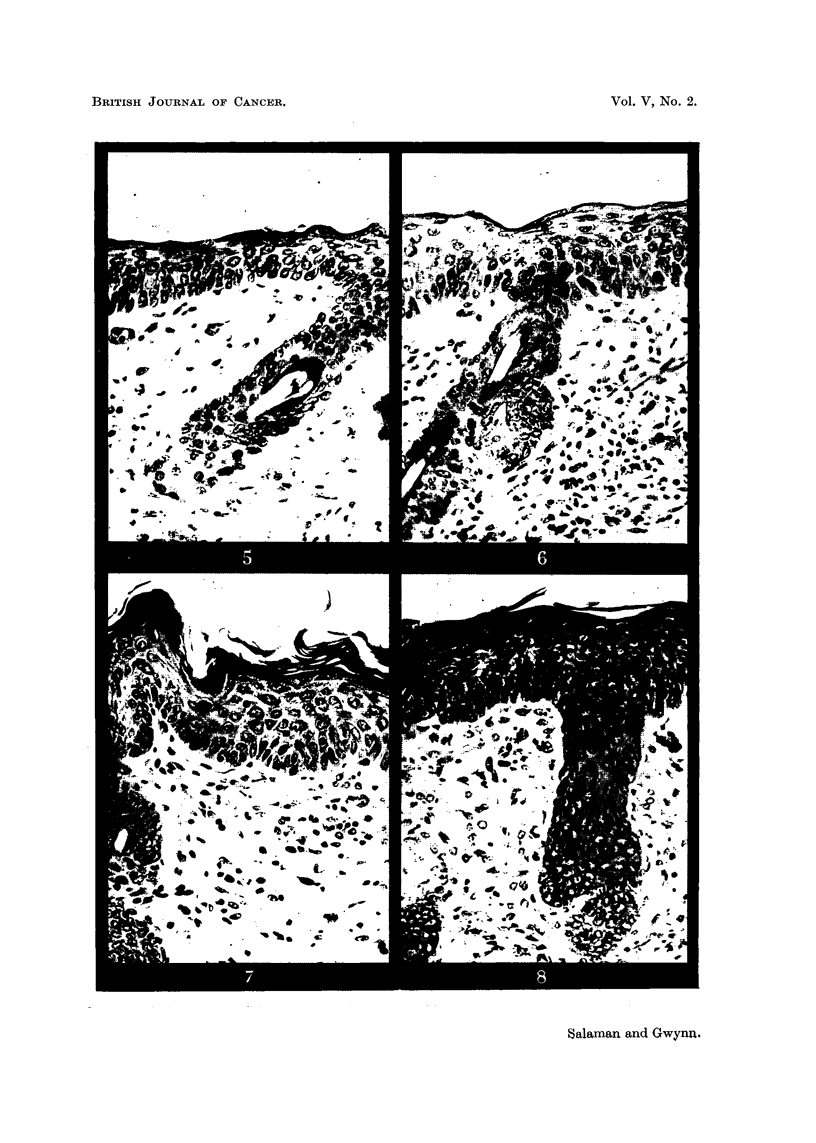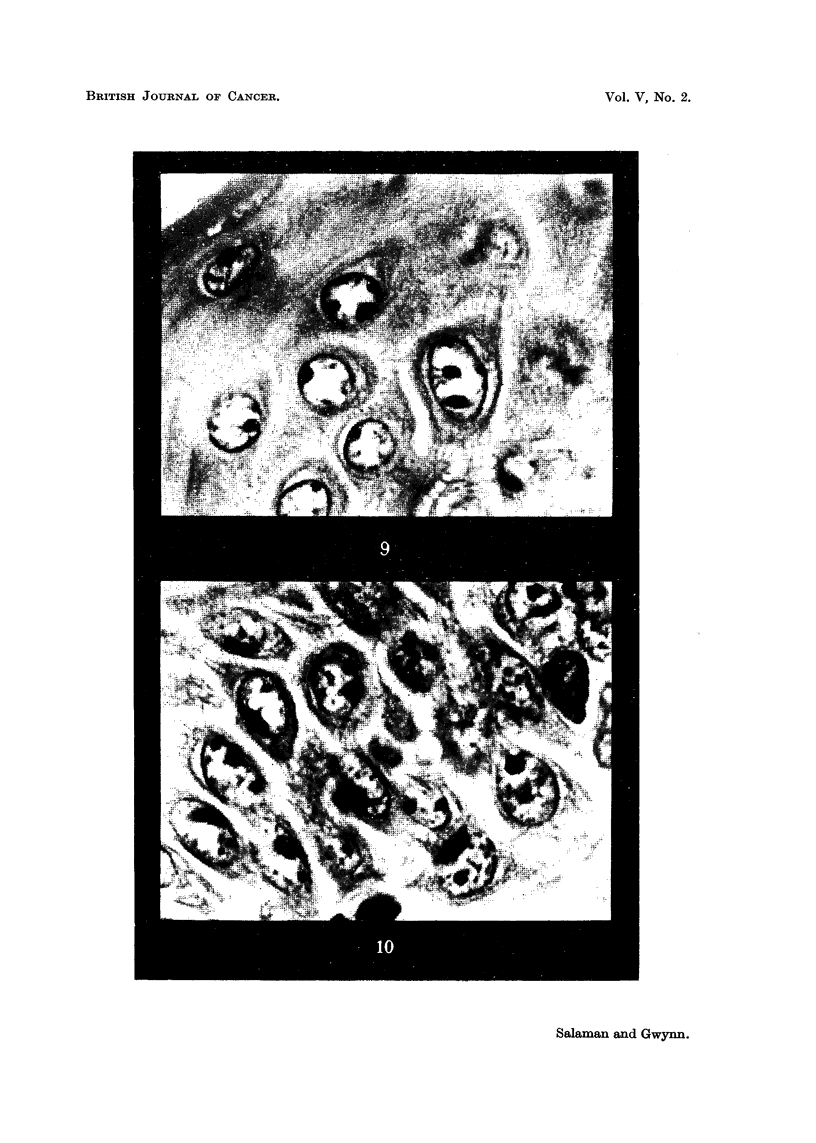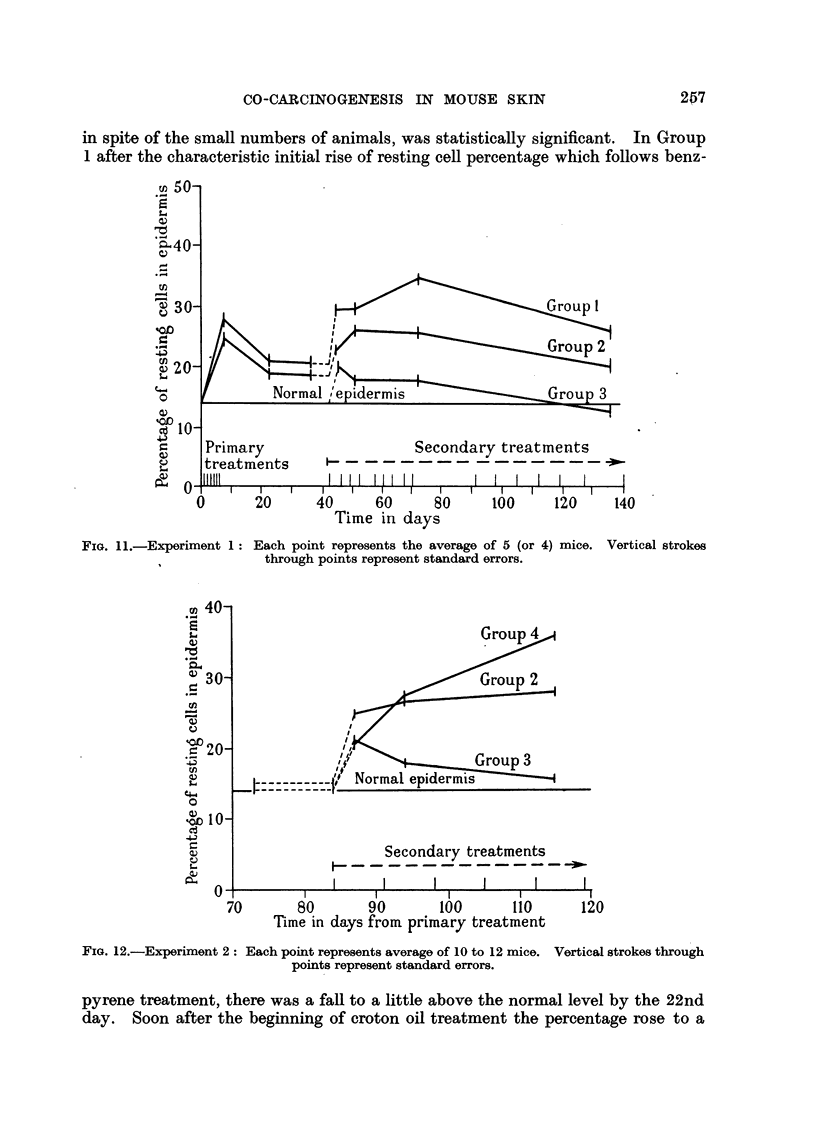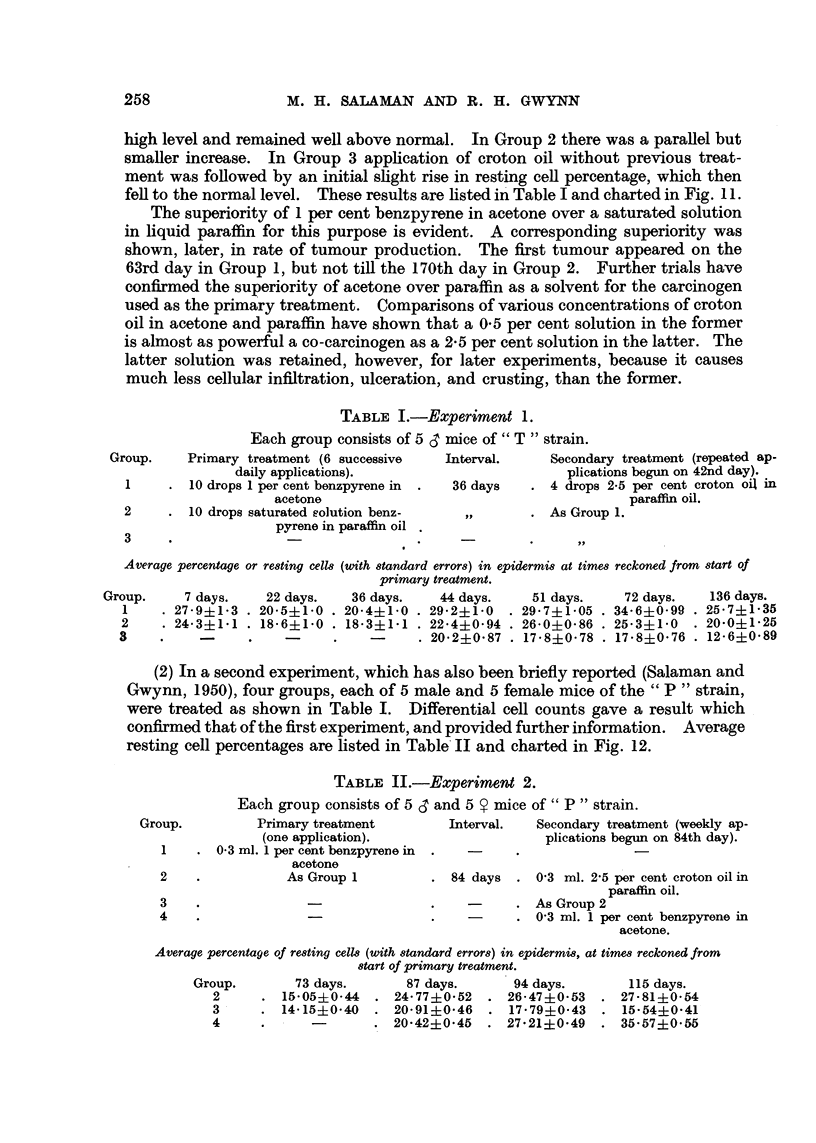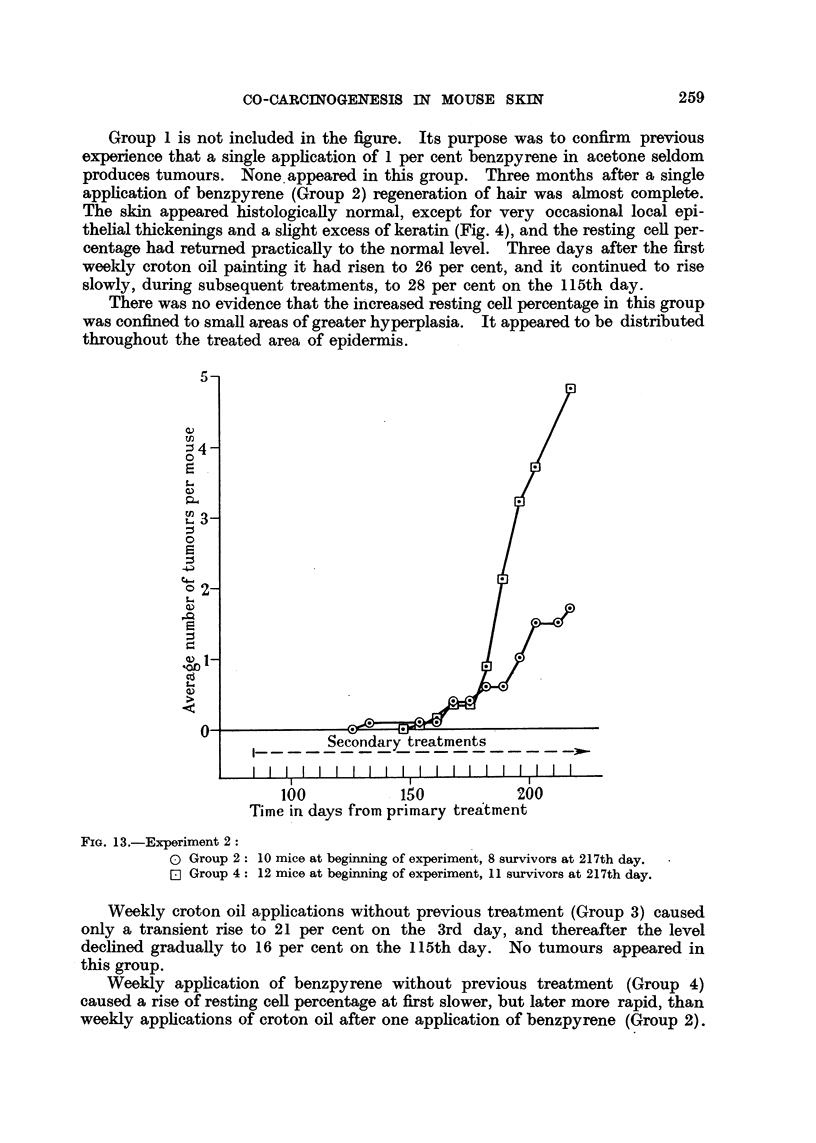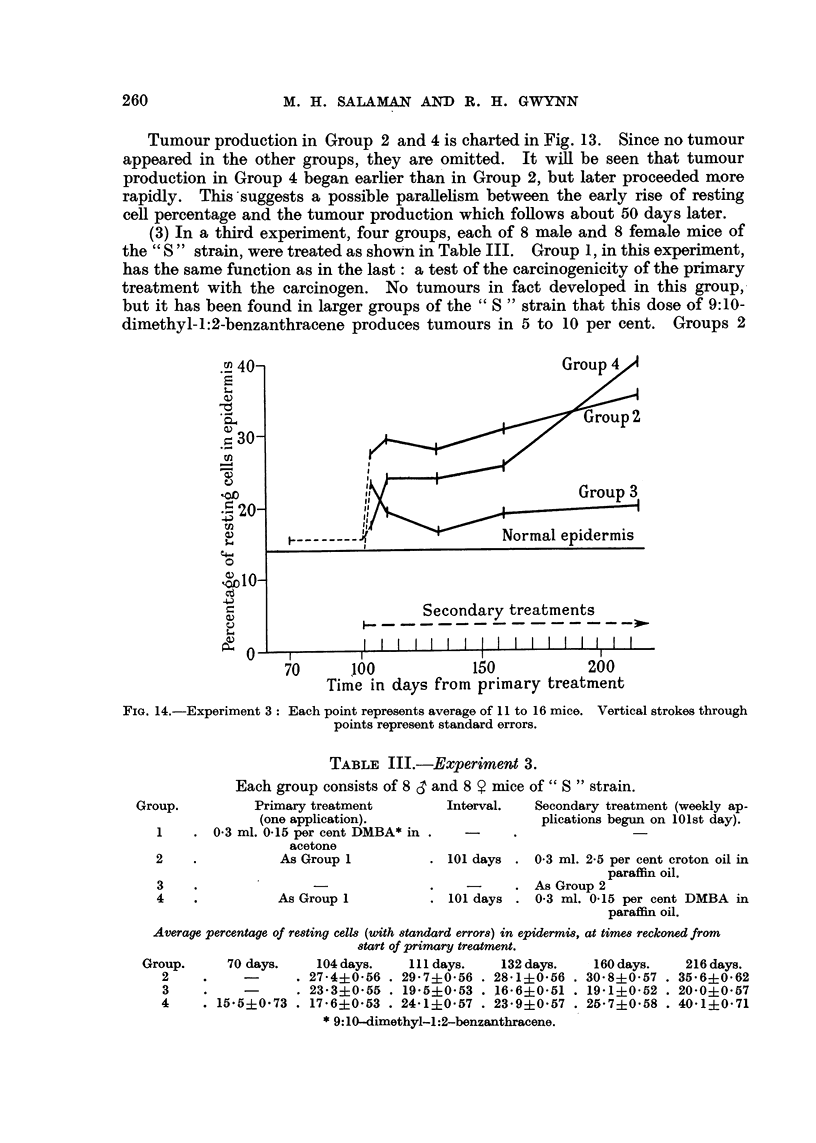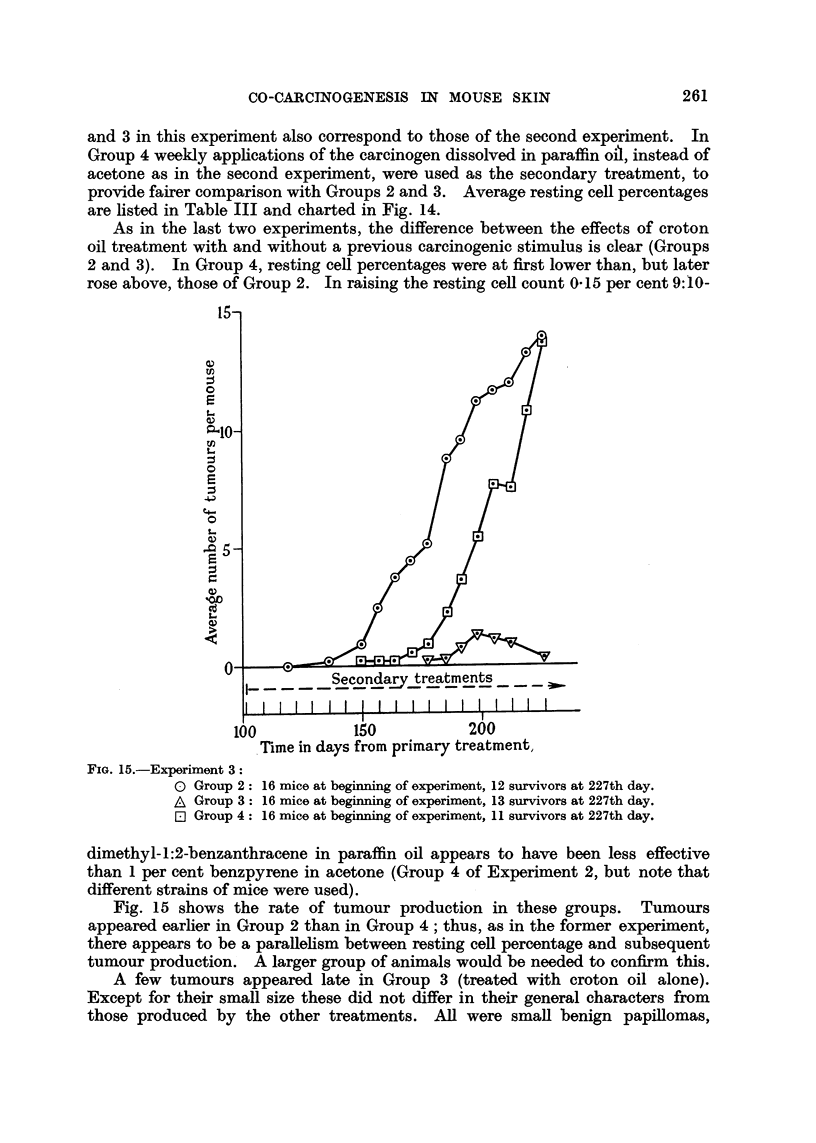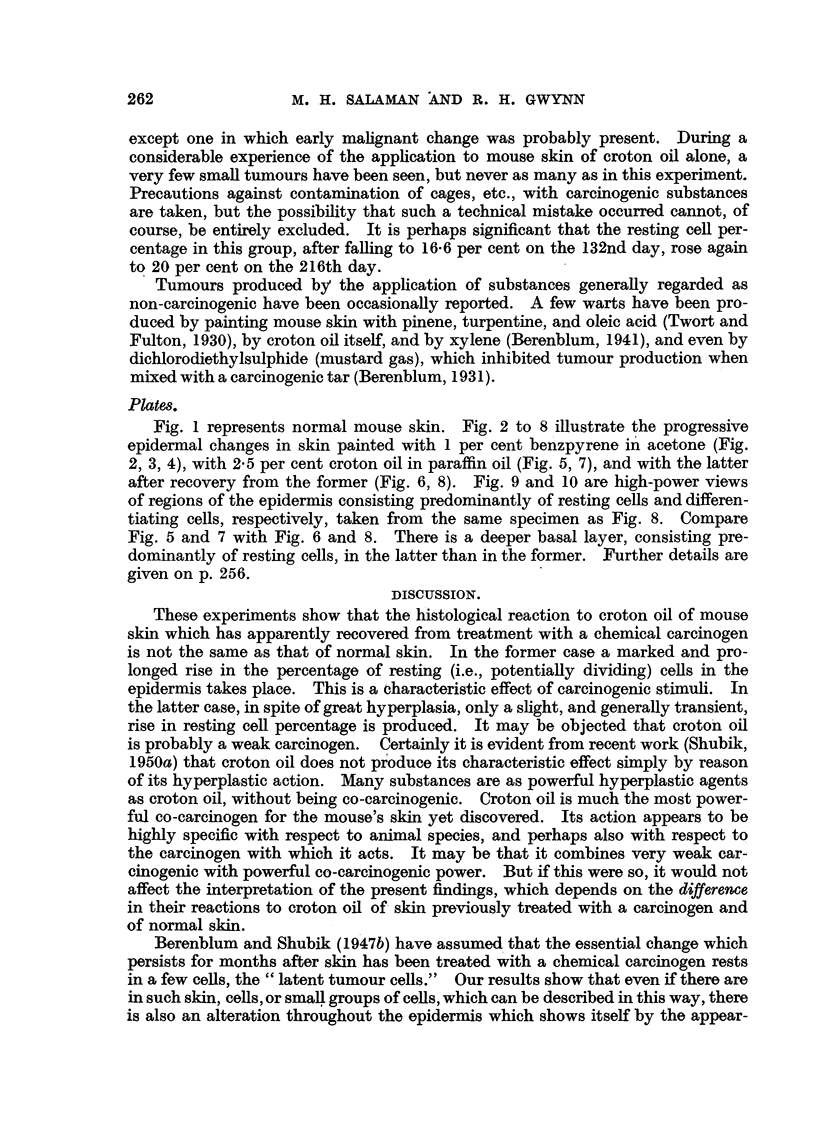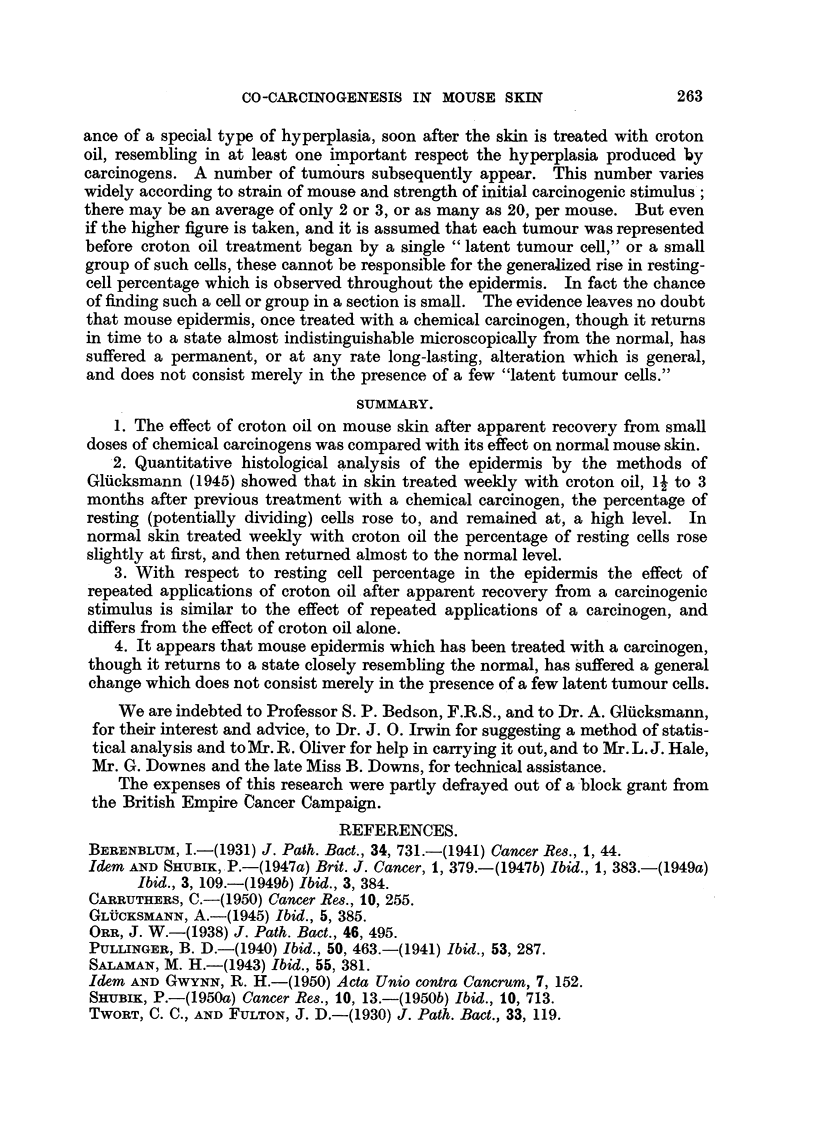# The Histology of Co-Carcinogenesis in Mouse Skin

**DOI:** 10.1038/bjc.1951.26

**Published:** 1951-06

**Authors:** M. H. Salaman, R. H. Gwynn

## Abstract

**Images:**


### THE HISTOLOGY OF CO-CARCINOGENESIS IN MOUSE SKIN.

M. H. SALAMAN AND R. H. GWYNN.

*From the Cancer Research Department, London Hospital Medical College, London, E. 1.*

Received for publication May 10, 1951.

THERE is still some disagreement on the question whether the early changes produced in epidermis by chemical carcinogens are specific, i.e., whether they can be distinguished from the effects of non-carcinogenic irritants. The chemical evidence is inconclusive. Cowdry and his school have been able to show that the chemical constituents of mouse epidermis made hyperplastic by several applications of 20 methylcholanthrene differ quantitatively from those of normal epidermis (Carruthers, 1950), but it does not appear to have been proved that these changes are produced only by carcinogenic substances. Histological evidence is more definite. The literature on the histogenesis of chemically-induced tumours of mouse skin was summarized by Glücksmann (1945). The present authors take the view, based on the qualitative histological observations of Pullinger (1940, 1941) and the quantitative differential cell counts of Glücksmann (1945), that the reaction of mouse epidermis to chemical carcinogens is recognizably different from its reaction to non-carcinogenic irritants, a view which has been strengthened by their own observations. Changes in the dermis following application of chemical carcinogens to the skin of mice have been described by Orr (1938), which are characteristic, though probably not so sharply specific, as those visible in the epidermis. Only the epidermal changes concern us in the present investigation.

Berenblum and Shubik (1947*a*, 1947*b*, 1949*a*, 1949*b*) have shown that when mouse skin is given a primary treatment with a carcinogen, insufficient by itself to produce tumours, and then, after an interval which may be as long as 43 weeks, is treated repeatedly with the non-carcinogenic irritant croton oil, tumours appear, and their number increases up to a maximum. This maximum depends on the concentration of the carcinogen used in the initial treatment, and on its potency when several carcinogens are compared, but, Berenblum and Shubik (1947*b*, 1949*b*) claim, is largely independent of the interval between the primary and the beginning of the secondary treatments. These experiments give a convincing demonstration of what has seemed probable from many observations of carcinogenesis in man and animals, namely, that the reaction of the skin to a carcinogenic stimulus is not uniform. Though the whole treated area reacts specifically, there must be, within that area, cells, or groups of cells, which suffer a change different in kind or in extent from that suffered by their neighbours. Berenblum and Shubik (1947*b*) have called these "latent tumour cells." They regard the reaction of skin to carcinogenic stimuli, apart from the formation of these cells, as an incidental accompaniment of tumour production.

If mouse skin is examined microscopically at intervals after a single application of a chemical carcinogen, the characteristic initial epidermal hyperplasia gradually disappears. The skin returns in about 3 months to a condition in which no abnormality can be detected microscopically, except a few slight thickenings and irregularities of the surface epithelium, slight thickening of the keratin layer, and dilatation of the mouths of some of the hair follicles (Fig. 4). The process may be practically complete in 6 weeks. The only significant way in which such skin has hitherto been found to differ from untreated skin is in its ability to produce tumours when treated with a co-carcinogen such as croton oil. The "latent tumour cells," if present, must be so few that they are very unlikely to be detected in sections, or perhaps they may not be recognizable if seen. The skin has passed through a state known to be produced only by carcinogenic agents ; this state has apparently passed away, yet the skin will then react to treatment with a non-carcinogenic substance in a manner quite different from normal skin.

The early reaction of normal mouse skin to croton oil is similar in general type to its reaction to other non-carcinogenic irritants, and differs in important respects from the reaction to carcinogenic substances. The chief criteria on which this distinction is based have been fully described by Pullinger (1940) and by Glücksmann (1945), and only a summary will be given here. Both carcinogenic and non-carcinogenic irritants, applied to mouse skin in suitable (i.e., non-necrotic and non-vesicating) concentrations, rapidly produce marked epithelial hyperplasia. Between the 2nd and 5th day the hyperplasia in the carcinogen-treated skin is very disorderly ; the average size of the epidermal cells is increased, with great individual variations, hair follicles are shortened and their mouths closed by keratin plugs, and sebaceous glands are undergoing squamous metaplasia and fusion with the follicles (Fig. 2). At this period skin treated with croton oil also shows marked epithelial hyperplasia, but the cells are more regular in size and arrangement ; there is epilation, but less damage to hair follicles or sebaceous glands (Fig. 5). Later, the two types of hyperplasia are more difficult to distinguish on these grounds (Fig. 3, 7). However, when the epidermal cells are classified by Glücksmann's method (1945), which is described below, definite differences are readily observable throughout the period of treatment. This method consists of the classification of all nucleated cells of the epidermis, excluding hair follicles, under 4 headings. These may be defined as follows (Glücksmann, 1945, with slight modifications) :

(a) *Resting cells*, i.e., basal cells proper, that are capable of division and have not yet embarked on keratinization. They are recognized by their large deeply-staining nuclei, sparse foamy basophilic cytoplasm, and ill-defined cell-boundaries (Fig. 10).

(b) *Differentiating cells*, i.e., cells of the *stratum spinosum* and *granulosum*. These have more distinct cell walls, greater amounts of more condensed and eosinophilic cytoplasm, and more lightly staining nuclei, than the resting cells; in the *stratum granulosum* they have keratohyalin granules (Fig. 9).

(c) *Mitotic cells* of all stages from prophase to telophase.

(d) *Degenerating cells*, i.e., cells in the process of nuclear pyknosis, karyorrhexis, or karyolysis.

Mention of the epidermal strata in the above descriptions refers to hyperplastic mouse skin, or to the normal skin of the foot, the tail, and the ear, where the epidermis is stratified. In the normal skin of the body of the mouse there are no strata, and the various types of cell are arranged apparently at random in a layer one cell, or in places two cells, thick.

Of the nucleated cells in the epidermis of the back of a normal adult mouse, about 14 per cent are resting, 80 per cent differentiating, and the rest are mitotic or degenerating.

This classification was used because it gives definite information about the state of the epidermis which is not obtainable by any other method known to us. This information is not dependent on any hypothesis about the function or potentialities of the different classes of cells. For the purpose of this work they are simply types of cells which can be distinguished, the absolute and relative numbers of which vary in different states of the skin.

One of the most striking features of the hyperplasia produced by chemical carcinogens in mouse epidermis is the rise in absolute and relative numbers of the resting cells. Non-carcinogenic irritants, on the other hand, produce a hyperplasia in which the absolute number of all epidermal cells is increased but their relative numbers remain approximately the same as in normal skin (Glücksmann, 1945). The percentage of resting cells in the epidermis was chosen as a criterion for comparison of the hyperplasia produced by the different treatments used in the following experiments. It provides a measure, which can be handled statistically, of the specificity of the reaction of the epidermis, i.e., of its resemblance to the characteristic reaction to carcinogenic substances.

In the work now reported the effect of croton oil on normal mouse skin has been compared histologically with its effect on skin after the reaction to previous treatment with a chemical carcinogen has apparently disappeared. Three strains of mice and two different carcinogens were used. The skin was examined histologically at intervals during treatment, and the differences found were given numerical expression in terms of the differential cell count.

### MATERIALS AND METHODS.

*Mice.*—Stock albino mice from three sources were used. "T" and "S" mice were purchased from dealers. "P" mice were of an institute-bred strain originally obtained from the National Institute for Medical Research; they had been used before for studies on skin carcinogenesis by Glücksmann (1945) and Salaman (1943). The reaction of all 3 strains to the carcinogens used, and to the croton oil, was satisfactory, though it varied in degree. They were fed on rat cubes made according to a formula recommended by the Rowett Institute, with a small addition of oats, and water *ad libitum.*

*Carcinogens.*—1:2-Benzpyrene and 9:10-dimethyl-1:2-benzanthracene were obtained from Messrs. L. Light & Co. Croton oil was obtained from Messrs. Boots Pure Drug Co.

*Solvents.*—Acetone was obtained from British Drug Houses (Analar grade), and liquid paraffin from British Drug Houses or Messrs. Allen & Hanbury. The grade of paraffin used had a specific gravity of 0·835–0·850. It was found to have practically no fluorescence when illuminated by U.V. light, and was thin enough to spread readily over the skin. Further notes on the choice of solvents and concentration of solutions are given in the descriptions of individual experiments.

*Methods of application.*—The hair of the back was clipped before the first treatment. Except where otherwise stated, 0·3 ml. amounts of the solutions were applied from volumetric or calibrated dropping pipettes to the whole of the back from the forelimbs to the root of the tail. The acetone solutions spread rapidly over this area ; the solutions in paraffin were gently spread over it with a glass spreader.

*Histological examination.*

At intervals during treatment small oval pieces of skin, about 1 cm. long and 0·5 cm. broad, were removed from the treated areas, under mixed ether and chloroform anaesthesia, fixed in Zenker's fluid, dehydrated in alcohol, cleared in cedar-wood oil, embedded in paraffin wax, cut at 8$\mu$, and stained with Ehrlich's haematoxylin followed by a mixture of eosin and Biebrich scarlet. For photographic purposes Heidenhain's iron haematoxylin without counterstain was used. General histological characters were noted, and differential counts of all nucleated epidermal cells, excluding hair follicles, were carried out by Glücksmann's method (1945) described above. In the first experiment all the cells in 1 mm. lengths of epidermis were counted in each of 3 alternate serial sections of each biopsy specimen, giving totals which varied from 300 to 600 cells. In the second experiment 200 cells in each of 4 alternate serial sections, making totals of 800, were counted. As this number was found to be greater than was necessary for statistical significance, in the third experiment 200 cells in each of 3 alternate serial sections, giving totals of 600, were counted. Averages and standard errors were calculated for each group of mice.

The method of calculating standard errors was as follows :

If $n$ = number of mice in each group ;
$N$ = total number of cells counted in each biopsy specimen ;
$R$ = number of resting cells " " " "
$P$ = percentage of resting cells " " "

The percentage of resting cells in each group at any one time is given by—

$$\frac{\Sigma R}{\Sigma N} = \bar{P}$$

and

$$\bar{Q} = 1 - \bar{P}$$

For each group at each time the value of

$$\frac{\Sigma\left(\frac{R^2}{N}\right) - \frac{(\Sigma R)^2}{\Sigma N}}{\bar{P}\bar{Q}}$$

is calculated.

If $\chi^2$ is the sum of the values of this expression for all groups at all times, and $D = \Sigma(n - 1)$ is the number of degrees of freedom for the whole experiment, then the standard error of $\bar{P}$ is given by—

$$\sqrt{\frac{\bar{P}\bar{Q}}{N}\left\{\left(\frac{\frac{\chi^2}{D} - 1}{n}\right) + \frac{1}{n}\right\}} = \sqrt{\left\{\frac{\bar{P}\bar{Q}}{Nn} \cdot \frac{\chi^2}{D}\right\}}.$$

Some practice is needed before the differential cell count gives reproducible results. An observer must to some extent establish his own standard of judgment in distinguishing the different types of cell, particularly resting and differentiating cells. When this has been done the counts are closely reproducible. It is advisable that all counts to be compared should be made by the same person. One of us (R. H. G.) has made all the counts in the present work. In order to detect the possible influence of unconscious bias all the slides in the third experiment (some 350) had their labels covered until the counts had been made. The standard errors of means were of the same order in this experiment as in the other two.

The mice were examined weekly for the appearance of tumours, for periods of 200 to 250 days. No distinction was made, in recording the numbers of tumours, between papillomas and epitheliomas. Shubik (1950*a*, 1950*b*) has recently shown that tumours produced by one application of a carcinogen followed by repeated applications of croton oil are predominantly benign. Our observations confirm his, though we have obtained a rather higher proportion of malignant tumours by this method than he did.

### EXPERIMENTS.

(1) In a preliminary experiment 3 groups, each of 5 young male adult mice of the " T " strain, were treated as follows :

*Group* 1.—(*a*) For 6 successive days 10 drops of a 1 per cent solution of benzpyrene in acetone applied daily to the whole skin of the back.

(*b*) Interval of 36 days.

(*c*) For 3 weeks 4 drops of a 2·5 per cent solution of croton oil in liquid paraffin applied twice a week to the same area, then, following an interval of 3 weeks (made necessary by severe crusting), once a week for a further 4 weeks.

*Group* 2.—(*a*) Ten drops of a saturated solution of benzpyrene in liquid paraffin (approximately 0·8 per cent) applied as in Group 1 (*a*).

(*b*) and (*c*) as in Group 1.

*Group* 3.—Croton oil solution applied as in Group 1 (*c*), without previous treatment.

Biopsies of treated skin were taken at intervals. The results of resting cell counts in the epidermis of Groups 1 and 3 have been briefly reported (Salaman and Gwynn, 1950). There was a definite difference between these groups which,

---

### EXPLANATION OF PLATES.

FIG. 1–10 : Biopsies of mouse skin, fixed Zenker's fluid, stained Heidenhain's haematoxylin.

FIG. 1.—Normal mouse skin from scapular region. × 285.

FIG. 2, 3.—Mouse skin 3 and 10 days respectively after the beginning of weekly applications of 1 per cent benzpyrene in acetone. × 285.

FIG. 4.—Mouse skin 75 days after one application of 1 per cent benzpyrene in acetone. × 285.

FIG. 5, 7.—Mouse skin 3 and 10 days respectively after the beginning of weekly applications of 2·5 per cent croton oil in paraffin oil. × 285.

FIG. 6, 8.—Mouse skin 3 and 10 days respectively after the beginning of weekly applications of 2·5 per cent croton oil in paraffin oil, following 84 days after one application of 1 per cent benzpyrene in acetone. × 285.

FIG. 9.—High-power view of a part of the epidermis shown in Fig. 8, where differentiating cells predominate. × 1700.

FIG. 10.—Another part of the same specimen as that shown in Fig. 8, where resting cells predominate. × 1700.

1
2
3
4

5 6 7 8

Salaman and Gwynn.

9

10

in spite of the small numbers of animals, was statistically significant. In Group 1 after the characteristic initial rise of resting cell percentage which follows benz-

FIG. 11.—Experiment 1 : Each point represents the average of 5 (or 4) mice. Vertical strokes through points represent standard errors.

FIG. 12.—Experiment 2 : Each point represents average of 10 to 12 mice. Vertical strokes through points represent standard errors.

pyrene treatment, there was a fall to a little above the normal level by the 22nd day. Soon after the beginning of croton oil treatment the percentage rose to a high level and remained well above normal. In Group 2 there was a parallel but smaller increase. In Group 3 application of croton oil without previous treatment was followed by an initial slight rise in resting cell percentage, which then fell to the normal level. These results are listed in Table I and charted in Fig. 11.

The superiority of 1 per cent benzpyrene in acetone over a saturated solution in liquid paraffin for this purpose is evident. A corresponding superiority was shown, later, in rate of tumour production. The first tumour appeared on the 63rd day in Group 1, but not till the 170th day in Group 2. Further trials have confirmed the superiority of acetone over paraffin as a solvent for the carcinogen used as the primary treatment. Comparisons of various concentrations of croton oil in acetone and paraffin have shown that a 0·5 per cent solution in the former is almost as powerful a co-carcinogen as a 2·5 per cent solution in the latter. The latter solution was retained, however, for later experiments, because it causes much less cellular infiltration, ulceration, and crusting, than the former.

TABLE I.—*Experiment* 1.

Each group consists of 5 ♂ mice of " T " strain.

| Group. | Primary treatment (6 successive daily applications). | Interval. | Secondary treatment (repeated applications begun on 42nd day). |
|---|---|---|---|
| 1 | 10 drops 1 per cent benzpyrene in acetone | 36 days | 4 drops 2·5 per cent croton oil in paraffin oil. |
| 2 | 10 drops saturated solution benzpyrene in paraffin oil | ,, | As Group 1. |
| 3 | — | — | ,, |

*Average percentage or resting cells (with standard errors) in epidermis at times reckoned from start of primary treatment.*

| Group. | 7 days. | 22 days. | 36 days. | 44 days. | 51 days. | 72 days. | 136 days. |
|---|---|---|---|---|---|---|---|
| 1 | 27·9±1·3 | 20·5±1·0 | 20·4±1·0 | 29·2±1·0 | 29·7±1·05 | 34·6±0·99 | 25·7±1·35 |
| 2 | 24·3±1·1 | 18·6±1·0 | 18·3±1·1 | 22·4±0·94 | 26·0±0·86 | 25·3±1·0 | 20·0±1·25 |
| 3 | — | — | — | 20·2±0·87 | 17·8±0·78 | 17·8±0·76 | 12·6±0·89 |

(2) In a second experiment, which has also been briefly reported (Salaman and Gwynn, 1950), four groups, each of 5 male and 5 female mice of the " P " strain, were treated as shown in Table I. Differential cell counts gave a result which confirmed that of the first experiment, and provided further information. Average resting cell percentages are listed in Table II and charted in Fig. 12.

TABLE II.—*Experiment* 2.

Each group consists of 5 ♂ and 5 ♀ mice of " P " strain.

| Group. | Primary treatment (one application). | Interval. | Secondary treatment (weekly applications begun on 84th day). |
|---|---|---|---|
| 1 | 0·3 ml. 1 per cent benzpyrene in acetone | — | — |
| 2 | As Group 1 | 84 days | 0·3 ml. 2·5 per cent croton oil in paraffin oil. |
| 3 | — | — | As Group 2 |
| 4 | — | — | 0·3 ml. 1 per cent benzpyrene in acetone. |

*Average percentage of resting cells (with standard errors) in epidermis, at times reckoned from start of primary treatment.*

| Group. | 73 days. | 87 days. | 94 days. | 115 days. |
|---|---|---|---|---|
| 2 | 15·05±0·44 | 24·77±0·52 | 26·47±0·53 | 27·81±0·54 |
| 3 | 14·15±0·40 | 20·91±0·46 | 17·79±0·43 | 15·54±0·41 |
| 4 | — | 20·42±0·45 | 27·21±0·49 | 35·57±0·55 |

Group 1 is not included in the figure. Its purpose was to confirm previous experience that a single application of 1 per cent benzpyrene in acetone seldom produces tumours. None appeared in this group. Three months after a single application of benzpyrene (Group 2) regeneration of hair was almost complete. The skin appeared histologically normal, except for very occasional local epithelial thickenings and a slight excess of keratin (Fig. 4), and the resting cell percentage had returned practically to the normal level. Three days after the first weekly croton oil painting it had risen to 26 per cent, and it continued to rise slowly, during subsequent treatments, to 28 per cent on the 115th day.

There was no evidence that the increased resting cell percentage in this group was confined to small areas of greater hyperplasia. It appeared to be distributed throughout the treated area of epidermis.

FIG. 13.—Experiment 2 :
○ Group 2 : 10 mice at beginning of experiment, 8 survivors at 217th day.
□ Group 4 : 12 mice at beginning of experiment, 11 survivors at 217th day.

Weekly croton oil applications without previous treatment (Group 3) caused only a transient rise to 21 per cent on the 3rd day, and thereafter the level declined gradually to 16 per cent on the 115th day. No tumours appeared in this group.

Weekly application of benzpyrene without previous treatment (Group 4) caused a rise of resting cell percentage at first slower, but later more rapid, than weekly applications of croton oil after one application of benzpyrene (Group 2).

Tumour production in Group 2 and 4 is charted in Fig. 13. Since no tumour appeared in the other groups, they are omitted. It will be seen that tumour production in Group 4 began earlier than in Group 2, but later proceeded more rapidly. This suggests a possible parallelism between the early rise of resting cell percentage and the tumour production which follows about 50 days later.

(3) In a third experiment, four groups, each of 8 male and 8 female mice of the "S" strain, were treated as shown in Table III. Group 1, in this experiment, has the same function as in the last: a test of the carcinogenicity of the primary treatment with the carcinogen. No tumours in fact developed in this group, but it has been found in larger groups of the "S" strain that this dose of 9:10-dimethyl-1:2-benzanthracene produces tumours in 5 to 10 per cent. Groups 2

FIG. 14.—Experiment 3: Each point represents average of 11 to 16 mice. Vertical strokes through points represent standard errors.

TABLE III.—*Experiment* 3.

Each group consists of 8 ♂ and 8 ♀ mice of "S" strain.

| Group. | Primary treatment (one application). | Interval. | Secondary treatment (weekly applications begun on 101st day). |
|---|---|---|---|
| 1 | 0·3 ml. 0·15 per cent DMBA* in acetone | — | — |
| 2 | As Group 1 | 101 days | 0·3 ml. 2·5 per cent croton oil in paraffin oil. |
| 3 | — | — | As Group 2 |
| 4 | As Group 1 | 101 days | 0·3 ml. 0·15 per cent DMBA in paraffin oil. |

*Average percentage of resting cells (with standard errors) in epidermis, at times reckoned from start of primary treatment.*

| Group. | 70 days. | 104 days. | 111 days. | 132 days. | 160 days. | 216 days. |
|---|---|---|---|---|---|---|
| 2 | — | 27·4±0·56 | 29·7±0·56 | 28·1±0·56 | 30·8±0·57 | 35·6±0·62 |
| 3 | — | 23·3±0·55 | 19·5±0·53 | 16·6±0·51 | 19·1±0·52 | 20·0±0·57 |
| 4 | 15·5±0·73 | 17·6±0·53 | 24·1±0·57 | 23·9±0·57 | 25·7±0·58 | 40·1±0·71 |

* 9:10–dimethyl–1:2–benzanthracene.

and 3 in this experiment also correspond to those of the second experiment. In Group 4 weekly applications of the carcinogen dissolved in paraffin oil, instead of acetone as in the second experiment, were used as the secondary treatment, to provide fairer comparison with Groups 2 and 3. Average resting cell percentages are listed in Table III and charted in Fig. 14.

As in the last two experiments, the difference between the effects of croton oil treatment with and without a previous carcinogenic stimulus is clear (Groups 2 and 3). In Group 4, resting cell percentages were at first lower than, but later rose above, those of Group 2. In raising the resting cell count 0·15 per cent 9:10-

FIG. 15.—Experiment 3 :
○ Group 2 : 16 mice at beginning of experiment, 12 survivors at 227th day.
△ Group 3 : 16 mice at beginning of experiment, 13 survivors at 227th day.
□ Group 4 : 16 mice at beginning of experiment, 11 survivors at 227th day.

dimethyl-1:2-benzanthracene in paraffin oil appears to have been less effective than 1 per cent benzpyrene in acetone (Group 4 of Experiment 2, but note that different strains of mice were used).

Fig. 15 shows the rate of tumour production in these groups. Tumours appeared earlier in Group 2 than in Group 4 ; thus, as in the former experiment, there appears to be a parallelism between resting cell percentage and subsequent tumour production. A larger group of animals would be needed to confirm this.

A few tumours appeared late in Group 3 (treated with croton oil alone). Except for their small size these did not differ in their general characters from those produced by the other treatments. All were small benign papillomas, except one in which early malignant change was probably present. During a considerable experience of the application to mouse skin of croton oil alone, a very few small tumours have been seen, but never as many as in this experiment. Precautions against contamination of cages, etc., with carcinogenic substances are taken, but the possibility that such a technical mistake occurred cannot, of course, be entirely excluded. It is perhaps significant that the resting cell percentage in this group, after falling to 16·6 per cent on the 132nd day, rose again to 20 per cent on the 216th day.

Tumours produced by the application of substances generally regarded as non-carcinogenic have been occasionally reported. A few warts have been produced by painting mouse skin with pinene, turpentine, and oleic acid (Twort and Fulton, 1930), by croton oil itself, and by xylene (Berenblum, 1941), and even by dichlorodiethylsulphide (mustard gas), which inhibited tumour production when mixed with a carcinogenic tar (Berenblum, 1931).

*Plates.*

Fig. 1 represents normal mouse skin. Fig. 2 to 8 illustrate the progressive epidermal changes in skin painted with 1 per cent benzpyrene in acetone (Fig. 2, 3, 4), with 2·5 per cent croton oil in paraffin oil (Fig. 5, 7), and with the latter after recovery from the former (Fig. 6, 8). Fig. 9 and 10 are high-power views of regions of the epidermis consisting predominantly of resting cells and differentiating cells, respectively, taken from the same specimen as Fig. 8. Compare Fig. 5 and 7 with Fig. 6 and 8. There is a deeper basal layer, consisting predominantly of resting cells, in the latter than in the former. Further details are given on p. 256.

### DISCUSSION.

These experiments show that the histological reaction to croton oil of mouse skin which has apparently recovered from treatment with a chemical carcinogen is not the same as that of normal skin. In the former case a marked and prolonged rise in the percentage of resting (i.e., potentially dividing) cells in the epidermis takes place. This is a characteristic effect of carcinogenic stimuli. In the latter case, in spite of great hyperplasia, only a slight, and generally transient, rise in resting cell percentage is produced. It may be objected that croton oil is probably a weak carcinogen. Certainly it is evident from recent work (Shubik, 1950*a*) that croton oil does not produce its characteristic effect simply by reason of its hyperplastic action. Many substances are as powerful hyperplastic agents as croton oil, without being co-carcinogenic. Croton oil is much the most powerful co-carcinogen for the mouse's skin yet discovered. Its action appears to be highly specific with respect to animal species, and perhaps also with respect to the carcinogen with which it acts. It may be that it combines very weak carcinogenic with powerful co-carcinogenic power. But if this were so, it would not affect the interpretation of the present findings, which depends on the *difference* in their reactions to croton oil of skin previously treated with a carcinogen and of normal skin.

Berenblum and Shubik (1947*b*) have assumed that the essential change which persists for months after skin has been treated with a chemical carcinogen rests in a few cells, the " latent tumour cells." Our results show that even if there are in such skin, cells, or small groups of cells, which can be described in this way, there is also an alteration throughout the epidermis which shows itself by the appearance of a special type of hyperplasia, soon after the skin is treated with croton oil, resembling in at least one important respect the hyperplasia produced by carcinogens. A number of tumours subsequently appear. This number varies widely according to strain of mouse and strength of initial carcinogenic stimulus; there may be an average of only 2 or 3, or as many as 20, per mouse. But even if the higher figure is taken, and it is assumed that each tumour was represented before croton oil treatment began by a single " latent tumour cell," or a small group of such cells, these cannot be responsible for the generalized rise in resting-cell percentage which is observed throughout the epidermis. In fact the chance of finding such a cell or group in a section is small. The evidence leaves no doubt that mouse epidermis, once treated with a chemical carcinogen, though it returns in time to a state almost indistinguishable microscopically from the normal, has suffered a permanent, or at any rate long-lasting, alteration which is general, and does not consist merely in the presence of a few "latent tumour cells."

### SUMMARY.

1. The effect of croton oil on mouse skin after apparent recovery from small doses of chemical carcinogens was compared with its effect on normal mouse skin.

2. Quantitative histological analysis of the epidermis by the methods of Glücksmann (1945) showed that in skin treated weekly with croton oil, 1½ to 3 months after previous treatment with a chemical carcinogen, the percentage of resting (potentially dividing) cells rose to, and remained at, a high level. In normal skin treated weekly with croton oil the percentage of resting cells rose slightly at first, and then returned almost to the normal level.

3. With respect to resting cell percentage in the epidermis the effect of repeated applications of croton oil after apparent recovery from a carcinogenic stimulus is similar to the effect of repeated applications of a carcinogen, and differs from the effect of croton oil alone.

4. It appears that mouse epidermis which has been treated with a carcinogen, though it returns to a state closely resembling the normal, has suffered a general change which does not consist merely in the presence of a few latent tumour cells.

We are indebted to Professor S. P. Bedson, F.R.S., and to Dr. A. Glücksmann, for their interest and advice, to Dr. J. O. Irwin for suggesting a method of statistical analysis and to Mr. R. Oliver for help in carrying it out, and to Mr. L. J. Hale, Mr. G. Downes and the late Miss B. Downs, for technical assistance.

The expenses of this research were partly defrayed out of a block grant from the British Empire Cancer Campaign.

### REFERENCES.

Berenblum, I.—(1931) *J. Path. Bact.*, **34**, 731.—(1941) *Cancer Res.*, **1**, 44.
*Idem* and Shubik, P.—(1947*a*) *Brit. J. Cancer*, **1**, 379.—(1947*b*) *Ibid.*, **1**, 383.—(1949*a*) *Ibid.*, **3**, 109.—(1949*b*) *Ibid.*, **3**, 384.
Carruthers, C.—(1950) *Cancer Res.*, **10**, 255.
Glücksmann, A.—(1945) *Ibid.*, **5**, 385.
Orr, J. W.—(1938) *J. Path. Bact.*, **46**, 495.
Pullinger, B. D.—(1940) *Ibid.*, **50**, 463.—(1941) *Ibid.*, **53**, 287.
Salaman, M. H.—(1943) *Ibid.*, **55**, 381.
*Idem* and Gwynn, R. H.—(1950) *Acta Unio contra Cancrum*, **7**, 152.
Shubik, P.—(1950*a*) *Cancer Res.*, **10**, 13.—(1950*b*) *Ibid.*, **10**, 713.
Twort, C. C., and Fulton, J. D.—(1930) *J. Path. Bact.*, **33**, 119.